# Influence of enhanced ultraviolet-B radiation during rice plant growth on rice straw decomposition with nitrogen deposition

**DOI:** 10.1038/s41598-018-32863-8

**Published:** 2018-09-28

**Authors:** Guixiang Zhou, Feng Wei, Xiuwen Qiu, Xiaofeng Xu, Jiabao Zhang, Xiaomin Guo

**Affiliations:** 1 Poyang Lake Eco-economy Research Center, Jiujiang University, Jiujiang, 332005 China; 2 State Key Laboratory of Soil and Sustainable Agriculture, Institute of Soil Science, Chinese Academy of Sciences, Nanjing, 210008 China; 3 Jiangxi Agricultural University, Nanchang, 330045 China

## Abstract

Although straw decomposition is important for ecosystem fertility and carbon balance, influence of ultraviolet-B (UV-B) radiation and nitrogen (N) deposition on this process is unclear. In this study, UV-B-exposed rice straw was decomposed under different N addition treatments for 15 months to investigate the indirect effects of UV-B radiation on straw chemistry and direct effects of N deposition on decomposition. UV-B exposure during rice plant growth changed the rice straw chemical composition, increasing the concentrations of acid-insoluble fraction (AIF), acid-soluble fraction, and UV-B-absorbing compounds. High N content had a negative effect on decomposition of rice straw exposed to enhanced and ambient UV-B radiation. Both AIF concentration and FTIR peak intensities indicated that lignin in rice straw was selectively preserved following N addition and UV-B radiation, reducing straw decomposition rate, which corresponded to lower activities of lignin-degrading enzymes in the later stage of decomposition. Thus, enhanced UV-B radiation during rice plant growth produced more recalcitrant substrates (lignin) and N reacted with lignin to produce more resistant compounds, further decreasing straw decomposition rate. UV-B radiation during plant growth and N deposition inhibit litter decomposition in agroecosystem, and their effects should be considered when establishing biogeochemical models in response to global changes.

## Introduction

Litter decomposition is an important ecosystem process that affects carbon (C) and nutrients recycling. Despite several studies on litter decomposition^1^, there are still certain uncertainties on how this essential process is affected by global change drivers such as ultraviolet-B (UV-B) radiation and nitrogen (N) deposition. As these uncertainties impede the conclusions drawn in biogeochemical models on litter decomposition^2^, it is ecologically crucial for predicting and quantifying the consequences of enhanced UV-B radiation and anthropogenic N deposition in the ecosystem.

While numerous studies have investigated the influence of increased solar UV-B radiation on plants, especially on plant growth, production, DNA damage, and morphology^3–5^, fewer studies have examined the UV-B effects on the decomposition of plant litter, an important ecosystem process. Litter decomposition may be directly and indirectly influenced by increased UV-B radiation. Several studies that evaluated the direct effects of UV-B radiation on litter decomposition have demonstrated that enhanced UV-B exposure accelerated photodegradation of lignin and/or changed the community and activity of decomposers, ultimately affecting the litter decomposition rate^4,6^. However, only limited studies have assessed the indirect effects of UV-B radiation on litter decomposition. UV-B radiation can indirectly influence litter decomposition by changing the litter chemistry in the tissues of growing plants, including increase in N^3^, cellulose^4^, UV-B-absorbing compounds (AU)^7^, and lignin^4^ contents, and decrease in soluble carbohydrates and lignin/N ratio^6^. Nevertheless, some reports did not find any evidence of the influence of supplemental UV-B exposure during plant growth on litter chemistry and decomposition^5^. These inconsistent findings may be owing to the differences in the environmental conditions, UV-B dose, and plant species employed.

As a key global change driver, N deposition plays a vital role in litter decomposition process by altering the organic matter composition and enzymes activities of microbial decomposers^8–10^; however, it is still unclear whether this is a broad effect or a chemical-fractions-specific effect on straw. Increasing studies have demonstrated a decline in the total microbial biomass and microbial respiration across a range of terrestrial ecosystems under N deposition^11–13^, which may be owing to several mechanisms that could suppress microbial growth, such as decrease in pH leading to soil acidification, loss of cation nutrients resulting in aluminum toxicity, etc.^14^.

Interestingly, N addition inhibits the activity of oxidative enzymes associated with lignin degradation, such as phenol oxidase and peroxidase^8,15^, suggesting its ability to preserve specific chemical fractions in decomposing litter. Some studies have indicated that N deposition has significant effects on cellulolytic enzyme activities^16,17^. Furthermore, N addition has been reported to selectively preserve lignin relative to polysaccharides^14^. Although the microbial responses to N addition are clear, there is still a lack of evidence for lower decomposition of specific chemical fractions in decomposing litter following N deposition.

The effect of N deposition on straw decomposition is debatable. Some reports have shown that N deposition can increase soil N availability and change the C/N ratios of straw, resulting in higher straw decomposition rate^18,19^. However, some studies have indicated that high N deposition inhibited straw decomposition owing to the accumulation of polymers produced by the reaction between N and lignin^20^. A meta-analysis showed that externally added N negatively affected litter decomposition in general, and that the effects considerably varied^21^. These negative effects have been reported to often occur when the N deposition dose was high and the litter quality was low (high lignin concentration and lignin/N ratio)^22^. Besides, litter quality has also been demonstrated to change with UV-B exposure during plant growth^6^, and numerous studies have suggested that UV-B exposure increased the content of phenolic compounds such as lignin and tannins^6,7^. However, it is still unclear whether the change in litter quality induced by UV-B exposure during plant growth could influence litter decomposition under the condition of N deposition.

In subtropical China, the amount of N deposition from the atmosphere exceeds 30 kg N ha^−1^ yr^−1 ^^23^, which is predicted to increase in future. However, to the best of our knowledge, there is a lack of empirical evidence for the chemical changes in UV-B-exposed litter in response to N deposition. Thus, the aim of this study was to assess the indirect effects of solar UV-B radiation during rice plant growth on rice straw decomposition, determine whether UV-B radiation could influence the chemical composition of rice straw, and ascertain whether the effects of UV-B exposure vary with different levels of added N. The rice plants were grown under enhanced UV-B radiation and the resulting litter was decomposed under three stimulated N addition treatments (0, 30, and 60 kg N ha^−1^ yr^−1^) for 15 months. The indirect effects of UV-B exposure were evaluated by determining the changes in C, N, and chemical characteristics of straw and their influence on subsequent decomposition under N deposition condition. In addition, the variations in the chemical components and lignin-degrading enzymes during straw decomposition were investigated based on Fourier transform infrared spectroscopy (FTIR) spectra. The study hypothesized that (1) enhanced UV-B radiation during rice plant growth could alter rice straw chemistry and inhibit straw decomposition and (2) the effect of N deposition on rice straw decomposition could be modified by exposure to UV-B radiation during rice plant growth.

## Results

### Effects of UV-B on initial straw chemistry

UV-B exposure during rice plant growth significantly affected the initial straw chemical characteristics. The acid-insoluble fraction (AIF), acid-soluble fraction (ASF), C/N ratio, AIF/N ratio, and AU of rice straw exposed to enhanced UV-B treatment (UVB) were 19%, 9%, 20%, 51%, and 44% higher than those of rice straw exposed to ambient UV-B treatment (Ambient), respectively. In contrast, soluble phenolics (PHE), non-structural carbohydrates (NSCs), soluble proteins (PRO), and N content of UVB were 4%, 15%, 42%, and 21% lower than those of Ambient, respectively (Table 1). However, lipids (LIP) and lignocellulose index (LCI) were unaffected by UV-B exposure (Table 1).

**Table 1:** Chemical characteristics (% of dry matter) and straw quality indices of the initial rice straw collected from Ambient and UVB treatments.

Chemical traits	Abbreviation	Ambient	UVB	*P*
Acid-insoluble fraction	AIF	12.73	15.16	<0.001
Acid-soluble fraction	ASF	35.46	38.58	<0.001
Soluble phenolics	PHE	16.61	16.02	<0.001
Lipids	LIP	8.95	8.82	0.230
Non-structural carbohydrates	NSCs	7.25	6.15	<0.001
Soluble proteins	PRO	2.68	1.55	0.001
Nitrogen	N	0.66	0.52	0.013
Carbon/N	C/N	60.00	72.00	<0.001
Acid-insoluble fraction /N	AIF/N	19.29	29.15	<0.001
UV-B-absorbing compounds	AU	0.34	0.49	0.003

### Residual mass

**Figure 1:**
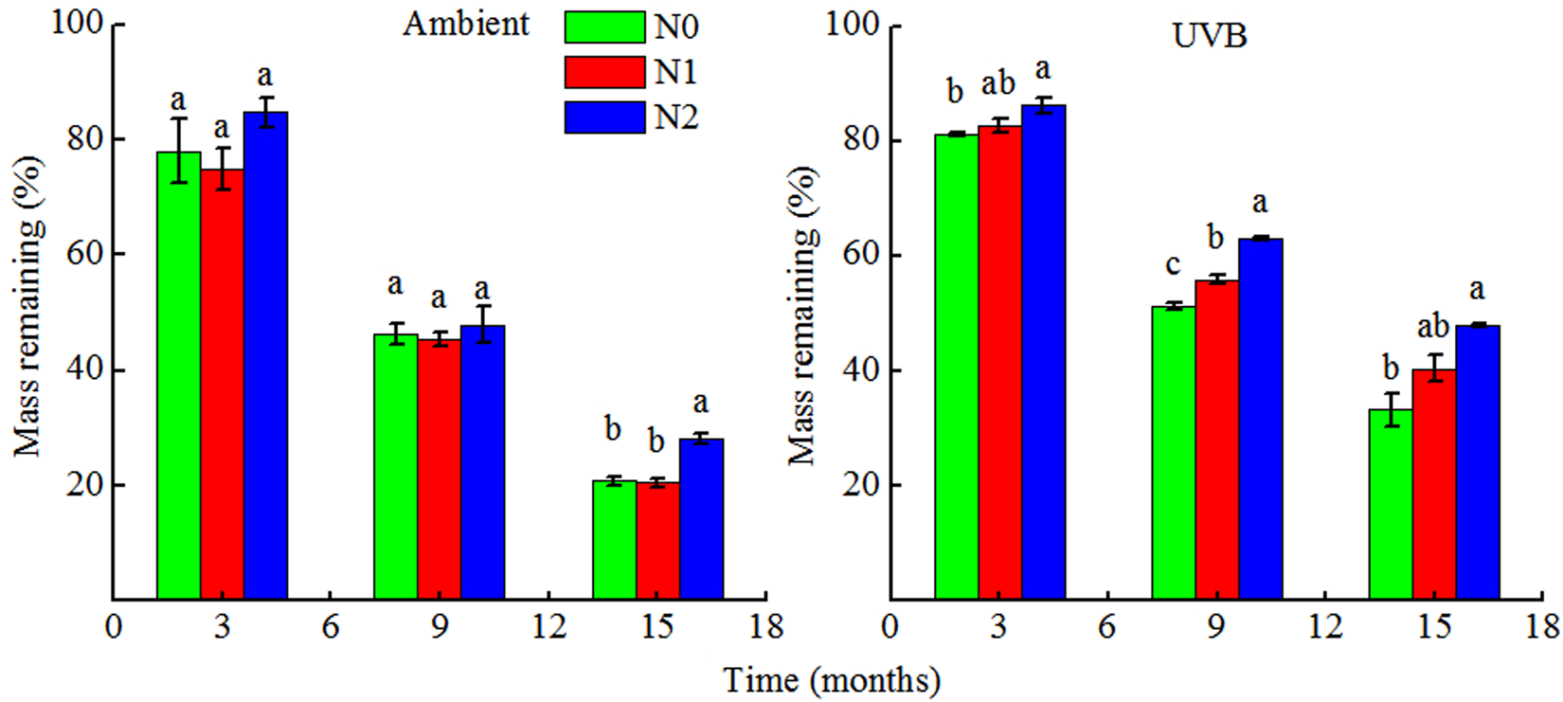
Fraction remaining of original mass in rice straw after 3, 9 and 15 months of decomposition with different N deposition treatments. Different lowercases indicate significant difference between N deposition treatments at each sample time (ANOVA with Tukey’s HSD, P ≤ 0.05). Ambient: ambient UV-B radiation during growth; UVB: enhanced UV-B radiation during growth; N0: control treatment without N addition; N1: 30 kg ha^−1^ yr.^−1^; N2: 60 kg ha^−1^ yr.^−1^

In general, the mass of rice straw was rapidly lost over the 15-month period. In Ambient, residual mass was unaffected by low N addition and did not present any statistical significance. In contrast, residual mass of Ambient under N2 (60 kg N ha^−1^ yr^−1^) treatment was the highest after 15 months of decomposition (Fig. 1, *P* < 0.05), indicating that high N content inhibited decomposition of rice straw under ambient solar radiation. The UV-B treatment during rice plant growth had a significant effect (*P* < 0.05) on rice straw decomposition process, and the UVB decomposed more slowly than the Ambient (Fig. 1). In particular, a significant effect of UV-B (*P* = 0.014) on rice straw decomposition was noted in UVB under N0 (0 kg N ha^−1^yr^−1^) treatment in 15 months (Fig. 1). Furthermore, in the UVB, a consistent pattern of increase in rice straw mass retention with increasing N deposition was observed in 3, 9, and 15 months of decomposition (Fig. 1).

### C and N dynamics

**Figure 2:**
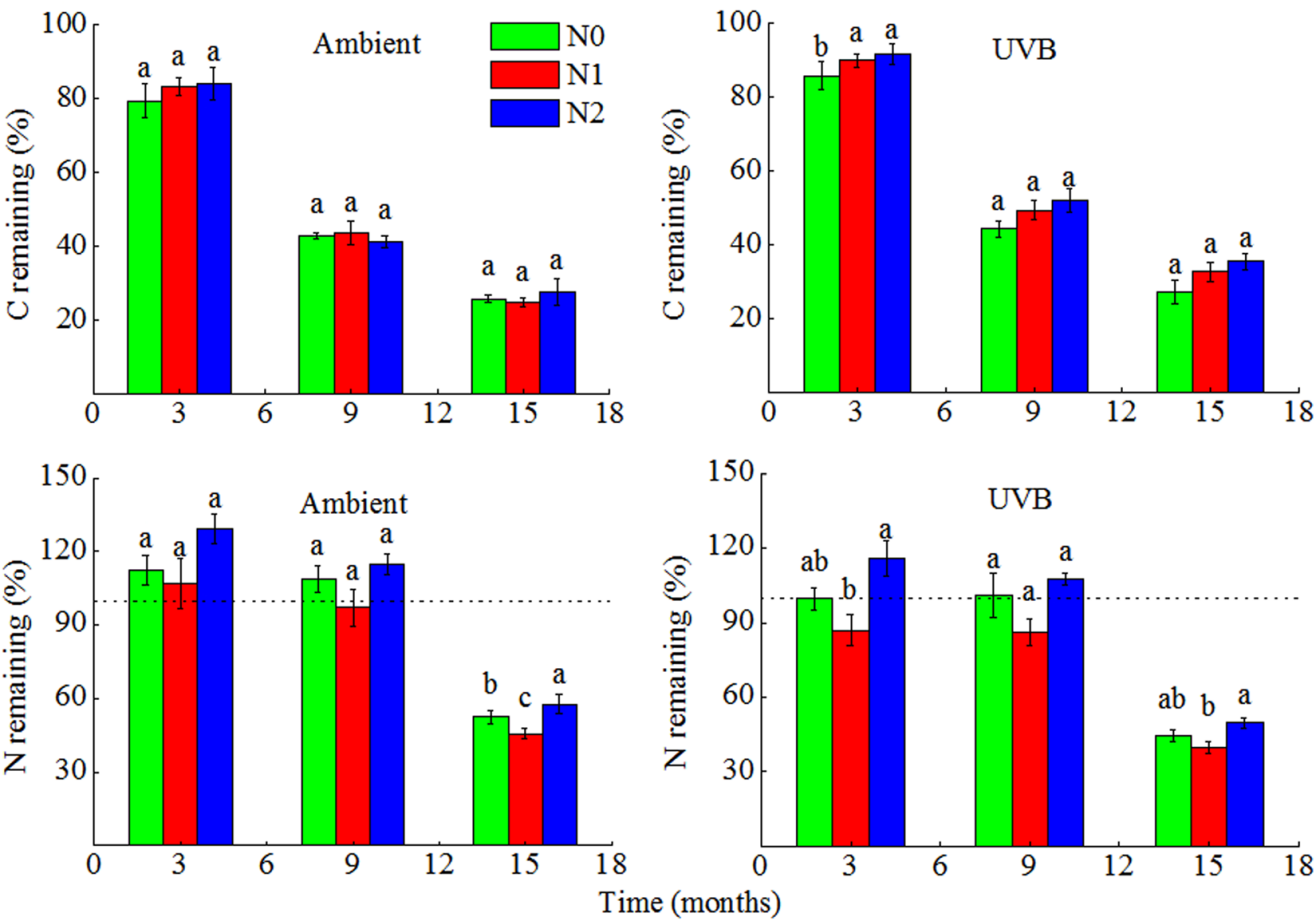
Fraction remaining of original C and N in rice straw after 3, 9 and 15 months of decomposition with different N deposition treatments. Different lowercases indicate significant difference between N deposition treatments at each sample time (ANOVA with Tukey’s HSD, P ≤ 0.05). Ambient: ambient UV-B radiation during growth; UVB: enhanced UV-B radiation during growth; N0: control treatment without N addition; N1: 30 kg ha^−1^ yr.^−1^; N2: 60 kg ha^−1^ yr.^−1^

The C content in Ambient under N0, N1, and N2 treatments declined to 26%, 25%, and 28% of that noted in the initial straw after 15 months of decomposition, respectively (Fig. 2), although no significant effect of N deposition on residual C content in rice straw was observed. With regard to N content, under all the N addition treatments, N was immobilized within 9 months of decomposition and the net N release from rice straw was obvious after 15 months of decomposition (Fig. 2). In the later stage of decomposition, low N addition (N1) treatment resulted in 13% decrease in residual N, whereas high N addition (N2) treatment caused 10% increase in residual N, when compared with that noted in N0 treatment (Fig. 2).

UV-B treatment produced minor effect on residual C, which was not statistically significant (Fig. 2), and the C content in UVB subjected to N0, N1, and N2 treatments declined to 27.22%, 32.86%, and 35.58% of that in the initial straw after 15 months of decomposition, respectively (Fig. 2). The residual N content during decomposition was slightly lower in UVB, when compared with that in Ambient (Fig. 2), and a similar pattern of N immobilization was observed in the first 9 months of decomposition in most of the UVB and Ambient. However, this N immobilization trend did not occur in the late stage of decomposition.

### Changes in straw chemistry during decomposition

During the initial decomposition (within 3 months) of Ambient, the residual NSCs, PHE, and LIP contents were 18.51%, 23.58%, and 60.40% of those observed initially before N treatment, respectively (Table 2). However, N addition did not affect the concentration of PHE and LIP after 15 months of decomposition. In the first 3 months of decomposition, N deposition significantly increased the contents of residual AIF and PRO in rice straw, but decreased the levels of residual NSCs and ASF in Ambient. However, these effects of N deposition on rice straw chemistry substantially disappeared in the late stage of decomposition (Table 2). The contents of AIF, PHE, and ASF were relatively higher and the NSCs and PRO fractions were lower in UVB, when compared with those in Ambient (Table 2). Furthermore, in the UVB, N addition led to a significant increase in residual AIF and PRO throughout the decomposition process, and an increase in residual ASF in the late stage of decomposition (Table 2).

**Table 2:** Fraction remaining (%) of chemical characteristics in rice straw after 3, 9 and 15 months of decomposition with different N deposition treatments.

Straw traits	Treatment	Ambient			UVB		
		3 months	9 months	15 months	3 months	9 months	15 months
AIF	N0	107.14 (0.71)	81.40 (5.18)	29.98 (1.70)	118.84 (0.65)	91.32 (2.51)	32.83 (0.40)
	N1	114.71 (1.54)	88.06 (3.90)	35.20 (1.19)	125.38 (0.84)	114.32 (9.22)	39.48 (0.62)
	N2	123.71 (1.60)	93.56 (7.26)	36.22 (1.90)	134.17 (1.48)	120.55 (3.45)	41.02 (0.87)
ASF	N0	80.33 (2.14)	41.97 (0.92)	26.02 (1.90)	85.44 (1.72)	56.28 (1.90)	30.82 (2.33)
	N1	75.28 (1.98)	40.81 (1.20)	22.38 (1.60)	81.81 (4.36)	53.70 (1.23)	26.84 (1.24)
	N2	70.96 (1.65)	34.11 (2.24)	18.88 (1.05)	75.39 (2.41)	49.26 (2.02)	21.69 (1.52)
PHE	N0	23.58 (1.47)	11.47 (1.67)	2.66 (0.28)	26.40 (1.40)	12.45 (0.40)	5.94 (0.47)
	N1	24.13 (0.77)	12.11 (0.32)	2.81 (0.30)	26.50 (0.99)	13.50 (0.99)	6.02 (0.25)
	N2	25.14 (0.94)	12.35 (0.82)	3.74 (0.54)	27.56 (1.65)	14.21 (0.75)	6.63 (0.40)
NSCs	N0	18.51 (0.66)	15.49 (0.54)	7.06 (0.38)	14.89 (0.87)	9.13 (0.39)	5.18 (0.36)
	N1	16.32 (0.53)	13.43 (0.54)	5.78 (0.65)	12.85 (0.76)	7.70 (0.53)	4.37 (0.39)
	N2	15.84 (0.51)	13.14 (0.42)	5.21 (0.28)	11.27 (0.83)	7.64 (0.64)	3.67 (0.28)
LIP	N0	60.40 (1.37)	35.69 (1.88)	19.95 (0.79)	59.69 (3.27)	33.51 (1.54)	19.03 (1.46)
	N1	60.57 (2.33)	36.57 (2.09)	20.34 (1.07)	61.36 (2.46)	34.14 (2.45)	19.77 (2.25)
	N2	64.28 (2.51)	39.20 (1.33)	22.07 (0.74)	63.01 (3.34)	37.20 (0.69)	22.27 (2.69)
PRO	N0	115.15 (0.88)	101.40 (3.33)	80.29 (3.82)	105.81 (0.86)	91.73 (4.97)	83.10 (5.97)
	N1	124.34 (3.32)	108.04 (4.36)	91.25 (3.96)	114.57 (0.86)	103.71 (2.15)	87.07 (3.87)
	N2	133.78 (2.57)	124.32 (3.49)	99.40 (6.00)	120.14 (1.83)	108.01 (1.26)	94.88 (5.46)

Values are means ± SE in parentheses (n = 3). AIF: Acid-insoluble fraction, ASF: Acid-soluble fraction, PHE: Soluble phenolics, NSCs: Non-structural carbohydrates, LIP: Lipids, PRO: Soluble proteins.

**Figure 3:**
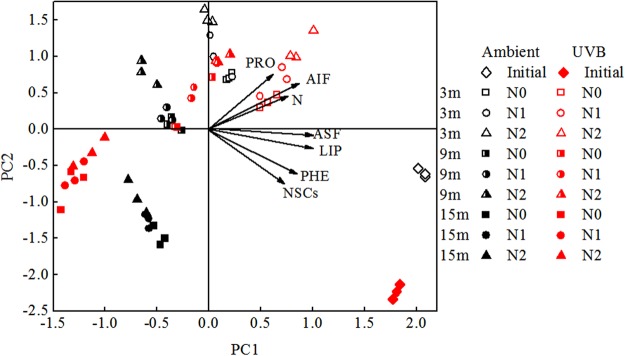
PCA based on the concentrations of major chemical characteristics. Two principal components explain a variance of 65.9% (PC1) and 27.0% (PC2). Ambient: ambient UV-B radiation during growth; UVB: enhanced UV-B radiation during growth; N0: control treatment without N addition; N1: 30 kg ha^−1^ yr.^−1^; N2: 60 kg ha^−1^ yr.^−1^; 3 m: 3 months, 9 m: 9 months, 15 m: 15 months.

Principle component analysis (PCA) was employed to assess the effects of UV-B and N deposition on the chemical characteristics of rice straw during decomposition. The PCA results showed that the points reflecting initial straw and decomposed straw samples were significantly separated (Fig. 3). In addition, the points denoting Ambient and UVB were obviously separated during the decomposition period. These findings indicated that UV-B radiation applied during rice plant growth was an important factor controlling the distribution of different chemical components of rice straw. The rice straw chemistry converged along PC1 axis, and AIF, ASF, and LIP presented longer arrows with PC1 axis. During the decomposition process, PC2 axis negatively correlated with ASF and LIP, and positively correlated with AIF (Fig. 3).

Pearson’s correlation analysis revealed that many of the chemical components in UVB and Ambient exhibited significant correlations with each other (Tables S1 and S2). In all the treatments, the changes in AIF were positively correlated with those in ASF, LIP, PRO, and N content (*P* < 0.001 for all treatments). Besides, a correlation was observed between PHE and N content in UVB (*P* = 0.021, Tables S1 and S2).

### FTIR analysis

**Figure 4:**
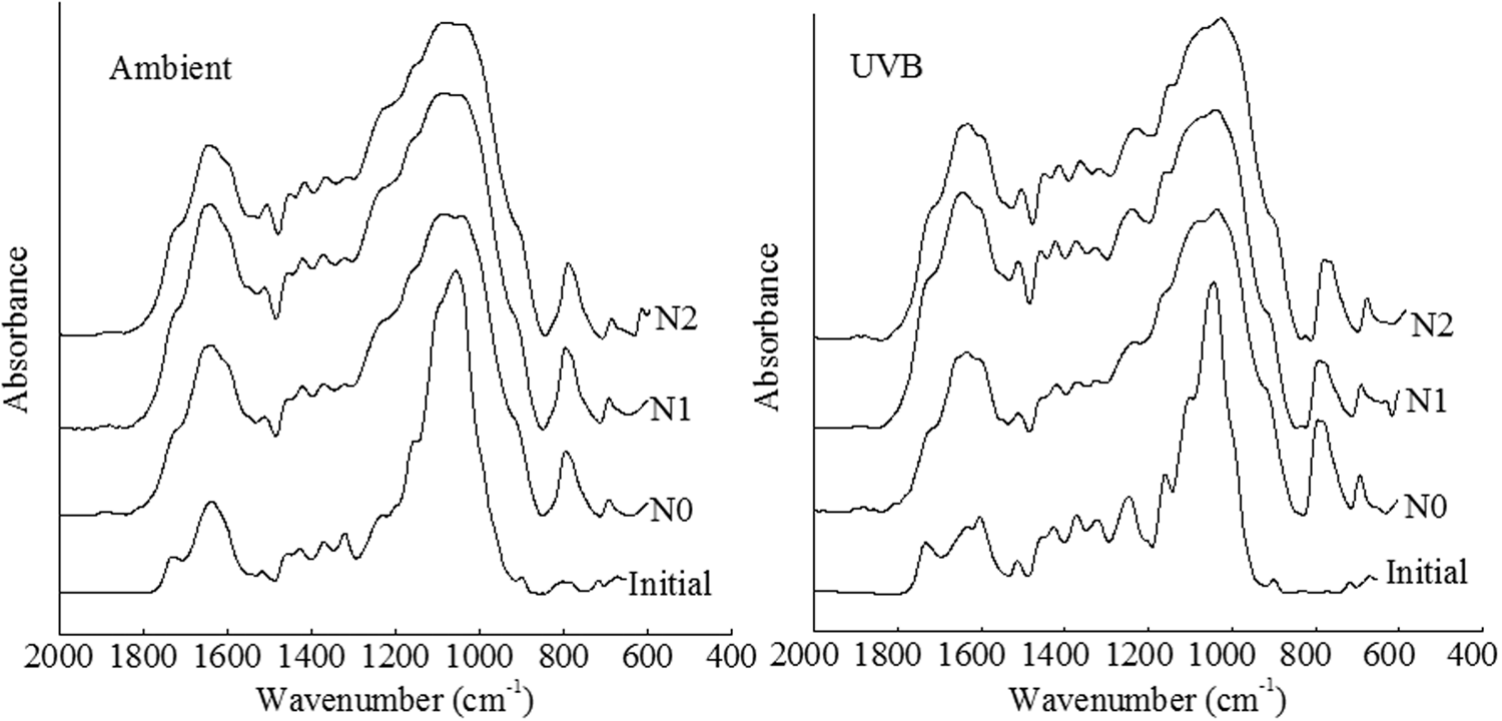
Fourier transform infrared spectroscopy of rice straw before decomposition and after 15 months of decomposition. Initial: rice straw before decomposition; Ambient: ambient UV-B radiation during growth; UVB: enhanced UV-B radiation during growth; N0: control treatment without N addition; N1: 30 kg ha^−1^ yr.^−1^; N2: 60 kg ha^−1^ yr.^−1^

FTIR has been widely used for identifying the characteristics of straws during the decomposition process. In the present study, the spectra for rice straw exhibited a number of distinct peaks in the fingerprint region ranging from 600 to 1800 cm^−1^ (Fig. 4). The lignin reference peak at 1510 cm^−1^ corresponded to aromatic skeleton vibration. The peak at around 898 cm^−1^ was identified to denote C-H deformation in cellulose. Prior to decomposition, the rice straw was dominated by the absorption of aromatic C = C (1596 cm^−1^) and intense absorption from 1100 to 1000 cm^−1^ (Fig. 4). During the 15-month rice straw decomposition process, the intensities of peaks at around 700–800 and 1650 cm^−1^ increased, while those of peaks at around 1738, 1320, and 898 cm^−1^ decreased (Fig. 4). An increase in the intensity and definition of peak at 1370, 1160, and 1510 cm^−1^ corresponding to C-H deformation in various polysaccharides, glyosidic linkage (C-O-C) vibration in polysaccharides, and aromatic skeleton vibration in lignin, respectively, was observed in UVB. In contrast, the intensity of peak at 1060 and 1650 cm^−1^ associated with C-O stretch in polysaccharides and quinines, respectively, obviously declined in UVB, when compared with that in Ambient in 15 months of decomposition (Fig. 4). The relative intensities of bands at around 1510 cm^−1^ (C = C bonds of aromatic rings) and 1650 cm^−1^ (quinones) were higher for rice straws subjected to N addition treatments, when compared with those for the control at the end of the experiment.

### Ligninolytic enzymes activities

**Figure 5:**
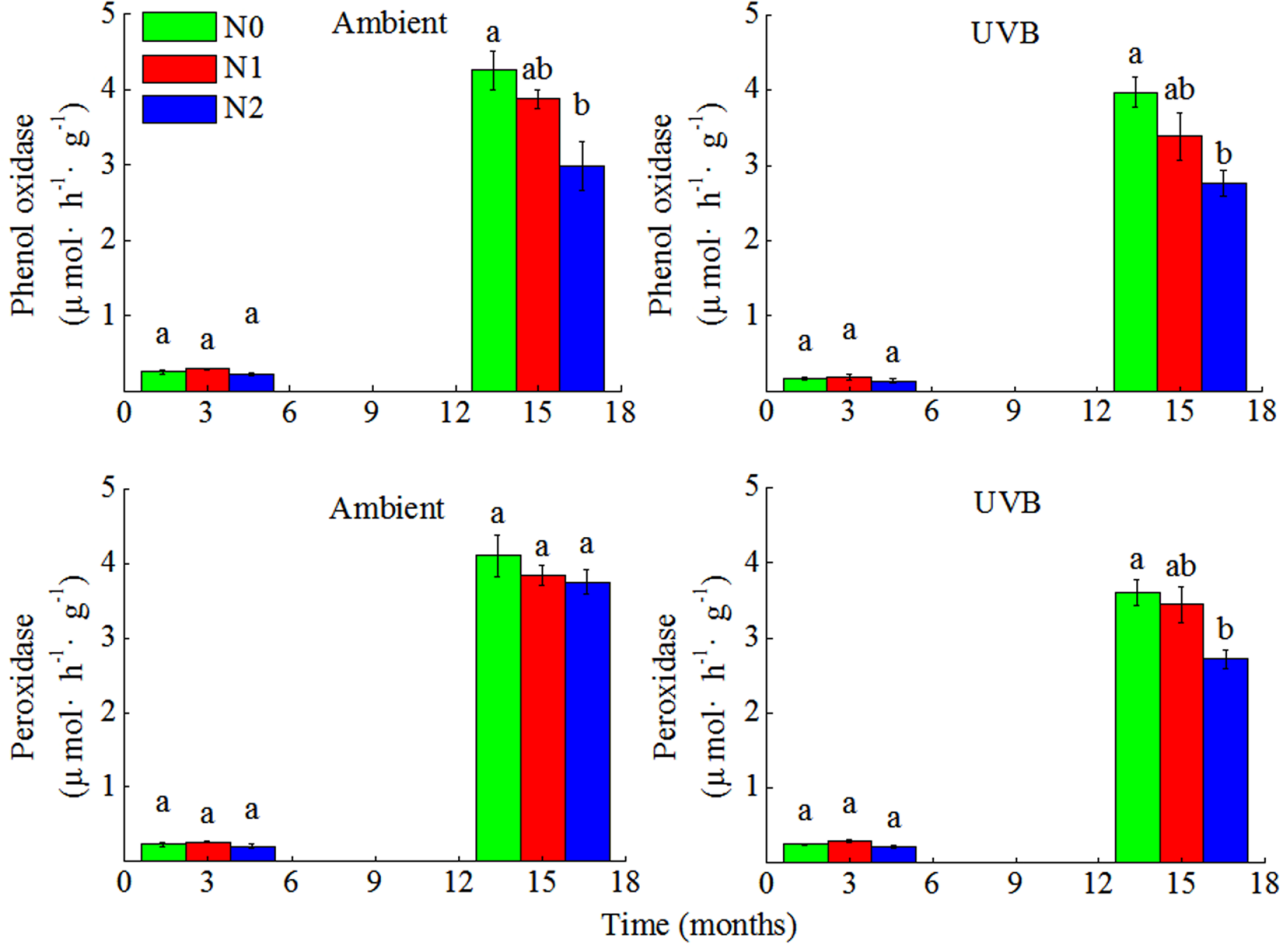
Activities of two lignin-degrading enzymes (phenol oxidase and peroxidase) during the 15 months of rice straw decomposition. Different lowercases indicate significant difference between N deposition treatments at each sample time (ANOVA with Tukey’s HSD, P ≤ 0.05). Ambient: ambient UV-B radiation during growth; UVB: enhanced UV-B radiation during growth; N0: control treatment without N addition; N1: 30 kg ha^−1^ yr.^−1^; N2: 60 kg ha^−1^ yr.^−1^

Both phenol oxidase and peroxidase activities were low and not significantly affected by N addition in UVB and Ambient in the early stage of decomposition (3 months, Fig. 5). However, after 15 months of decomposition, N addition declined the activity of phenol oxidase in both UVB and Ambient. Furthermore, high N addition significantly suppressed the activity of peroxidase in UVB in the later stage of decomposition (15 months), whereas no significant effect was noted in Ambient (Fig. 5).

## Discussion

Exposure to UV-B radiation during rice plant growth indirectly declined the decomposition rate of rice straw, when compared with that observed in the control, which is consistent with our first hypothesis. While UV-B exposure during rice plant growth did not significant affect the early stage of straw decomposition, it presented a significant (*P* < 0.05) negative effect on the later stage of straw decomposition (Fig. 1). These results are consistent with some, but not all, previous studies on the decomposition of litter exposed to UV-B exposure. For example, Pancotto *et al*.^6^ observed a similar indirect effect of UV-B exposure decreasing the decomposition rate of barley litter. However, some studies have demonstrated that exposure to UV-B radiation during growth increased the decomposition of *Quercus sp*.^5^ and *Triticum sp*. litter^3^, or did not affect the decomposition of litter^24^. These contradictory results could be owing to specific plant species responses or differences in the intensity of UV-B exposure and growth conditions^6^.

The effect of UV-B radiation during rice plant growth on the decomposition of rice straw might be the result of changes in the chemical composition in rice plant tissues. Exposure to UV-B radiation during rice plant growth increased the contents of AIF, ASF, C/N, AIF/N, and AU in the initial straw (Table 1), which might have resulted in an increase in the recalcitrant fractions that are difficult to break down by soil microorganisms, leading to a decline in rice straw decomposition rate. These changes in litter quality are consistent with those reported in a previous study that showed that UV-B radiation during plant growth resulted in increased production of AU, such as lignin and tannin, which suppress the growth of some fungal species^7^. During decomposition, the breakdown of soluble carbohydrates is easier than that of lignin and cellulose by microorganisms^7,14^. Therefore, in the present study, the concentrations of lignin were higher, whereas the contents of NSCs were lower in the UVB, when compared with those in the Ambient, during decomposition. These results confirmed that the differences in the initial straw chemistry between UVB and Ambient persisted throughout the process of straw decomposition. Furthermore, variations in the concentrations of resistant substrates such as lignin were observed between the UVB and Ambient during straw decomposition (Table 2, Figs 3 and 4). The peak near 1510 cm^−1^ is often employed as the characteristic lignin peak. The FTIR spectra for the UVB showed that the initial rice straw exhibited a strong peak intensity near 1510 cm^−1^, when compared with those for the Ambient (Fig. 4), thus affirming the above-mentioned results. The activities of microbial decomposers may be limited in UVB with higher lignin content^24^, ultimately resulting in lower decomposition rate, when compared with those in Ambient (Fig. 1). These findings are consistent with those reported in other studies. For instance, Pancotto *et al*.^24^ compared the decomposition of *Gunnera* litter (relatively high lignin content) and barley litter (relatively low lignin content) under UV-B condition, and illustrated that the microbial decomposition was lower when lignin content was high.

Furthermore, in the present study, exposure to UV-B radiation during rice plant growth also influenced N accumulation and release in rice straw (Fig. 1), and the residual N content in UVB was lower than that in Ambient. In contrast, Pancotto *et al*.^6^ reported that barley litter exposed to UV-B radiation released less N, when compared with that subjected to reduced UV-B treatment. These discrepancies in the results could be attributed to the differences in the initial litter quality or UV-B radiation intensity during plant growth.

In addition to the indirect effects of UV-B exposure during plant growth, N deposition is also known to influence litter decomposition. A meta-analysis of decomposition demonstrated that externally supplied N often presented negative relationships with decomposition^21^. In the present study, low N addition did not affect rice straw decomposition, whereas high N deposition inhibited the decomposition process (Table 2). This result is consistent with the finding reported by Knorr *et al*.^21^, who demonstrated that the negative effect of N on decomposition increased with increasing N addition rate of up to 20-fold of ambient N deposition. Inorganic N can react with lignin to form more resistant compounds during decomposition^20^. Furthermore, N limits microbial growth and suppresses the activities of enzymes associated with lignin degradation^8^. Therefore, it can be concluded that inhibition of lignin degradation by N addition contributes to the negative effects of high N deposition on litter decomposition.

The different responses of straw decomposition to N addition could be partly related to straw quality. Knorr *et al*.^21^ demonstrated that N addition decreased the mass loss in low-quality litters with high lignin concentrations, similar to that observed in the present study. AIF is a suitable indicator for lignin in litters, revealing that N addition selectively protected lignin, when compared with the rest of the straw mass^14^. The FTIR spectra obtained in the present study also showed that N addition increased the peak intensities at 1510 cm^−1^ (Fig. 4), indicating that lignin was preserved during decomposition. Therefore, the slow straw decomposition observed in the present study might have probably resulted from the suppression of AIF decomposition following N deposition, especially in UV-B-exposed straw with high lignin concentration.

The response of ligninolytic enzymes activities in decomposing straw appears to provide some explanations for the inhibitory effect of N addition on straw decomposition rate in all the N addition treatments. The suppression of lignin degradation by N addition has also been reported in other studies that implied that large amount of exogenous N inhibited microbial metabolism of lignin and suppressed lignin-degrading enzymes activities^20^. Phenol oxidase is an important lignin-degrading enzyme produced by white rot fungi and certain microorganisms belonging to Basidiomycota, Xylariaceae, and Ascomycota^25^, which was strongly inhibited by N addition in the UVB and Ambient in the later stage of straw decomposition in the present study (Fig. 5). These findings are in line with some previous reports, but are contradictory to certain studies. For instance, Freedman *et al*.^15^ found that the activities of phenol oxidase and peroxidase associated with lignin degradation decreased under simulated N deposition conditions. Hobbie *et al*.^20^ demonstrated a strong relationship between the negative effects of N on decomposition and the dynamics of ligninolytic enzymes activities in *Quercus ellipsoidalis* litter. On the contrary, some studies did not find any obvious effects of N addition on lignin-degrading enzymes activities in soil or litter^26^. Thus, the mechanisms related to the negative effects of N on the later stages of litter decomposition may vary with different sites and litter types owing to the variation in enzymes activities. Furthermore, the added N may react with C compounds such as polyphenols in litter to form other recalcitrant complexes, thus making the enzymes less effective under N-enriched condition.

The findings of the present study showed that exposure to UV-B during rice plant growth led to increased lignin and AU fraction, and a decline in soluble carbohydrates content. During decomposition of rice straw, excess N sourced from N addition may interact with lignin (partly formed from UV-B exposure during rice plant growth) to form recalcitrant compounds that are difficult to breakdown by microorganisms^5^. In addition, N addition could suppress the activity of lignin-degrading enzymes^26^, making it more difficult to decompose the accumulated lignin in UVB samples with added N. As a result, the residual mass of UV-B-exposed rice straw following 15 months of decomposition with N deposition was higher, supporting our second hypothesis. Thus, UV-B exposure during plant growth and N deposition during decomposition might be expected to function in the same direction, with more evident inhibitory effect on straw decomposition.

## Conclusion

UV-B exposure during rice plant growth changed the initial rice straw chemistry, increasing the concentrations of AIF, ASF, C/N, lignin/N, and AU, which may limit the activities of microbial decomposers, indirectly resulting in a decrease in the straw decomposition rate. The negative effects of high N deposition on straw decomposition in UVB and Ambient were observed in the later stage of decomposition partly owing to the decrease in lignin-degrading enzymes. The changes in the rice straw characteristics induced by UV-B radiation during rice plant growth aggravated the negative effects on straw decomposition under N deposition condition. Thus, UV-B radiation during plant growth and N deposition during litter decomposition may have incremental inhibitory effects on the dynamics of C pools in agroecosystem, and such effects should be more widely considered in biogeochemical models in future.

## Methods

### Study site

The study was conducted in Jiujiang city (29°68′N, 115°98′E), Jiangxi Province, China. The mean annual temperature and precipitation at the experimental site is 17 °C and 1407 mm, respectively. The area has a monsoonal subtropical climate with four distinct seasons and an annual average of 240 frost-free days. The main crop cultivated in this area is *Oryza sativa* (rice). The soil was derived from quaternary red clay, and is classified as Typic Plinthudult (Ultisols). The average pH, organic matter content, and soil total N content of the experimental site was 6.0, 11.4 g kg^−1^, and 1.3 g kg^−1^, respectively.

### Rice growth experiment under UV-B radiation

Rice seeds (*O. sativa* L.) were surface-disinfected with 5% NaClO and presoaked in distilled water. The seeds were then grown at 25 °C in a growth chamber with a light/dark cycle of 14/10 h for 20 days. Rice seedlings with similar heights were transplanted into pots (30 cm in diameter, 20 cm in height) containing 5 kg soil/pot. The pots were placed in greenhouse under two UV-B treatments: enhanced UV-B radiation (15%; UVB) and ambient UV-B radiation (or solar radiation; Ambient). Fertilizers N (as NH_4_HCO_3_) equivalent to 120 kg ha^−1^, P (as Ca(H_2_PO_4_)_2_·H_2_O) of 80 kg ha^−1^, and K (as KCl) of 80 kg ha^−1^ were added to each pot to provide essential nutrients for seedling growth. The UV-B radiation (280–320 nm) received during plant growth was manipulated using fluorescent UV-B lamps (UV-B313EL, Beijing Lighting Research Institute, Beijing, China) and plastic filters. The lamps were mounted on a metal frame and erected 30 cm above the rice seedlings. Aclar plastic film (Aclar Type 22 A film, 125-μm thickness, DuPont Co., Beijing, China), which allowed the transmission of 95% of solar radiation, was used for Ambient. UVB treatment was regulated using artificial irradiance from fluorescent UV-B lamps. The lamps were wrapped with cellulose acetate film (0.07 mm, DuPont Co., Beijing, China), which allowed transmission of both UV-A (320–400 nm, long wave) and UV-B (290–320 nm, medium wave), but removed all UV-C (200–290 nm, short wave). The UV-A transmission was not controlled. The films were replaced every 2 months to maintain acceptable limits of light transmission. The fluorescent UV-B lamps were operated for 8 h daily from 09:00 to 17:00 and regulated to maintain 15% enhancement above the ambient UV-B exposure until harvesting of rice. The UV-B energy was measured using a UV-297 radiometer (Photoelectric Instrument Factory of Beijing Normal University, Beijing, China).

### Rice straw decomposition experiment

In September 2015, the post-harvest rice straw was gathered from the experiment pots. The straw samples were air-dried for 1 month and oven-dried at 50 °C for 48 h in a dryer. The oven-dried straw samples were cut into 1 cm by using a straw chopper. The initial characteristics of UVB and Ambient are summarized in Table 1.

The rice straw decomposition rate was determined using nylon litterbag method. The litterbags were 20-cm long and 15-cm wide with a 1-mm mesh-size. Each litterbag filled with 15 g of oven-dried straw was placed on the surface of the soil in pots. The harvested rice straw was subjected to three N addition treatments, 0 kg N ha^−1^yr^−1^ (control; N0), 30 kg N ha^−1^yr^−1^ (low N; N1), and 60 kg N ha^−1^yr^−1^ (high N; N2), with three replicates. Based on the previous methods simulating N deposition^18^, ammonium nitrate (NH_4_NO_3_) dissolved in water was uniformly sprayed using a sprayer into the N1 and N2 pots at the end of each month. In the control (N0 pots), equivalent water without NH_4_NO_3_ was sprayed every month. The litterbags with rice straw under different N addition treatments were collected from each pot after 3, 9 and 15 months of decomposition, respectively. The soil particles were removed from the litterbags, and the rice straw samples were oven-dried at 50 °C to a constant weight in a dryer and weighted.

### Mass loss and chemical analysis

The residual mass of each rice straw sample was expressed as the percentage of initial straw dry weight. The first-order exponential decay model^27^, X_t_/X_0 = _e^-*k*t^, was used to calculate the annual decomposition rate constant *k* (yr^−1^), where X_t_ is the mass remaining at time t and X_0_ is the initial mass. The straw samples collected at each sampling time were ground and sieved. The total C and N contents were measured by using a CN analyzer (Elementar, Vario Micro Select, Germany).

The AIF, ASF, NSCs, soluble phenolics, soluble proteins, and total lipids contents in the rice straw samples were tested according to the methods of Xia *et al*.^14^. Briefly, the main chemical components were separated using a sequential extraction procedure. A two-phase H_2_SO_4_ hydrolysis was employed to separate the AIF and ASF. Phenol-sulfuric acid analysis was used to determine the sugars content representing NSCs. Folin-Ciocalteu reagent based on catechin standards was employed to test the content of soluble phenolics. Coomassie protein Bradford Reagent was used to determine the contents of proteins, and lipids were extracted with a mixture of chloroform and methanol solution.

After being oven-dried at 50 °C for 48 h, the rice straw samples were mixed with KBr, compressed, and analyzed with a Perkin Elmer 16 F PC Fourier transform infrared spectroscopy (FTIR) apparatus at 4000–400 cm^−1^. The region between 2000 and 600 cm^−1^ was examined because of the drastic change in the intensity of the peaks. The infrared spectra for the initial and decomposed straw samples (after 15 months of decomposition) were collected, and 64 scans at a resolution of 4 cm^−1^ were recorded for each sample. The spectra were base-line corrected^28^ and normalized using OMNIC software v9.0. The peak heights were determined against the baseline to represent the intensity of the bands of interest.

### Enzyme activity

During 15 months of rice straw decomposition, the straw samples were analyzed for the presence of oxidative enzymes associated with degradation of polyphenolic compounds, such as lignin, based on the methods of Saiya-Cork *et al*.^29^ and Sinsabaugh *et al*.^30^. The oxidative enzymes, phenol oxidase and peroxidase, were assayed using L-3,4-dihydroxyphenylalanine and hydrogen peroxide as substrates, respectively. In brief, approximately 0.5 g of the straw sample was homogenized in 125 ml of acetate buffer (50 mmol/L, pH 5.0) for 1 min, and the suspensions were dispensed into 96-well microplate. The activities of oxidative enzymes were determined as the absorbance on a microplate spectrophotometer (460 nm).

### Data and statistical analysis

Nutrients release during straw decomposition was expressed as the percentage of initial nutrients concentration, which was calculated by determining the nutrients concentration at each sampling time and dividing it by the initial nutrients concentration as follows: Residual nutrients (%) = 100 × [(M_t_ × C_t_)/ (M_0_ × C_0_)], where M_t_ is the mass at time *t*, C_t_ is the nutrients concentration at time *t*, M_0_ is the initial mass, and C_0_ is the initial nutrients concentration.

One-way analysis of variance (ANOVA) was employed to test the statistical significance of the differences in the annual decomposition rate, straw mass loss, and nutrients release at each sampling time, considering *P* < 0.05. To visualize the chemical shifts during decomposition, PCA was used to characterize the initial and decomposed rice straw chemistry at individual harvest points based on the concentrations of major chemical components. Pearson’s correlation analysis of the residual fractions of different chemical constituents and N concentration was performed to investigate how the chemical components interact with each other during decomposition. The values obtained in this study are expressed as mean ± standard error (n = 3). All the data were analyzed using SPSS 16.0 (Statistical Package for the Social Sciences) and figures were derived by using the software Origin 8.0.

## Electronic supplementary material

Supplementary information
