# Supplementary material for: Influence of enhanced ultraviolet-B radiation during rice plant growth on rice straw decomposition with nitrogen deposition
Source: Sci Rep. 2018 Sep 28;8:14512. doi: 10.1038/s41598-018-32863-8 (PMC6162212; doi:10.1038/s41598-018-32863-8)
Supplement: Supplementary file 1 — Supplementary information [file 41598_2018_32863_MOESM1_ESM.pdf]

Supplementary information

Influence of enhanced ultraviolet-B radiation during rice plant growth on rice straw decomposition with nitrogen deposition

Guixiang Zhou[1,2], Feng Wei[3], Xiuwen Qiu[1*], Xiaofeng Xu[3], Jiabao Zhang[2], Xiaomin Guo[3]

[1] Poyang Lake Eco-economy Research Center, Jiujiang University, Jiujiang 332005, China

[2] State Key Laboratory of Soil and Sustainable Agriculture, Institute of Soil Science, Chinese Academy of Sciences, Nanjing 210008, China

[3] Jiangxi Agricultural University, Nanchang 330045, China

[*]Corresponding author: Xiuwen Qiu

E-mail: qiuxiuwen5@163.com

Table S1. Pearson's correlation coefficient matrix for fraction remaining of straw chemical classes during 15 months of decomposition under Ambient conditions during growth.

Table S2. Pearson's correlation coefficient matrix for fraction remaining of straw chemical classes during 15 months of decomposition under UVB conditions during growth.

Table S1. Pearson's correlation coefficient matrix for fraction remaining of straw chemical classes during 15 months of decomposition under Ambient conditions during growth.

	AIF	ASF	PHE	LIP	NSC	PRO	N
AIF	1.00						
ASF	0.73***	1.00					
PHE	0.33	0.78***	1.00				
LIP	0.65 ***	0.95 ***	0.91 ***	1.00			
NSC	0.20	0.68 ***	0.98 ***	0.83 ***	1.00		
PRO	0.84 ***	0.48 ***	0.08	0.40*	-0.04	1.00	
N	0.90 ***	0.58 ***	0.26	0.55 **	0.16	0.78***	1.00

* $P < 0.05$, ** $P < 0.01$, *** $P < 0.001$.

Table S2. Pearson's correlation coefficient matrix for fraction remaining of straw chemical classes during 15 months of decomposition under UVB conditions during growth.

	AIF	ASF	PHE	LIP	NSC	PRO	N
AIF	1.00						
ASF	0.77***	1.00					
PHE	0.33	0.76***	1.00				
LIP	0.62***	0.93***	0.90***	1.00			
NSC	0.17	0.64***	0.98 ***	0.82***	1.00		
PRO	0.81***	0.56***	0.19	0.51**	0.03	1.00	
N	0.90 ***	0.75***	0.42*	0.63***	0.30	0.70***	1.00

* $P < 0.05$, ** $P < 0.01$, *** $P < 0.001$.
